# Toward Precision Feeding Regarding Minerals: What Is the Current Practice in Commercial Dairy Herds in Québec, Canada?

**DOI:** 10.3390/ani11051320

**Published:** 2021-05-05

**Authors:** Mélissa Duplessis, Liliana Fadul-Pacheco, Débora E. Santschi, Doris Pellerin

**Affiliations:** 1 Agriculture et Agroalimentaire Canada, Centre de Recherche et Développement de Sherbrooke, 2000 rue College, Sherbrooke, QC J1M 0C8, Canada; 2 Department of Animal and Dairy Sciences, University of Wisconsin, 1675 Observatory Drive, Madison, WI 53706, USA; lfpacheco@wisc.edu; 3 Lactanet, 555 Boul. des Anciens-Combattants, Sainte-Anne-de-Bellevue, QC H9X 3R4, Canada; dsantschi@lactanet.ca; 4 Département des Sciences Animales, Université Laval, 2425 rue de l’Agriculture, Québec, QC G1V 0A6, Canada; doris.pellerin@fsaa.ulaval.ca

**Keywords:** trace mineral, phosphorus, nutrient requirement, cross-sectional study, environment

## Abstract

**Simple Summary:**

It has been known for several years that limiting phosphorus in the cow diet mitigates its excretion in manure, hence reducing the environmental phosphorus load after manure spreading. The quantity of phosphorus that could be applied in the field is regulated by law in several countries. This is not the same for trace minerals such as cobalt, copper, manganese, and zinc. Nevertheless, if overfed, these last minerals are excreted in manure in great quantities and could accumulate in the soil after manure spreading, which could lead to detrimental environmental effects. However, formulating cow rations according to the mineral requirements is challenging for nutritionists. The aim of this analysis is to compare dietary phosphorus, cobalt, copper, manganese, and zinc concentrations from 100 commercial Holstein dairy herds with the National Research Council recommendations. Phosphorus is included as a point of comparison, as its overfeeding has been well studied compared with other studied trace minerals. The results indicate that, at the median, phosphorus, cobalt, copper, manganese, and zinc were respectively fed 8%, 405%, 52%, 372%, and 65% over the recommendations. This suggests that most nutritionists are aware that precision feeding regarding phosphorus is important for dairy production sustainability. It also shows that the other studied minerals were fed in excess and that some attempts should be made to reduce the mineral concentrations of diets.

**Abstract:**

This analysis is performed to obtain information on the current situation regarding phosphorus (P), cobalt (Co), copper (Cu), iron (Fe), manganese (Mn), and zinc (Zn) concentrations in cow diets of commercial dairy herds in Québec, Canada, and to compare them with National Research Council recommendations. Data are collected on 100 Holstein dairy herds in Québec, Canada, and 4430 cows were involved. Rations are analyzed for selected minerals and cow requirements relative to the recommendations were calculated. Median percentages of mineral recommendations fulfilled by forage were 55%, 196%, 54%, 776%, 181%, and 44% for P, Co, Cu, Fe, Mn, and Zn, respectively. Daily dietary concentrations of P, Cu, Mn, and Zn decreased as lactation progressed, whereas Co and Fe were stable throughout lactation. Phosphorus was the mineral fed the closest to the requirements, cows below 21 days in milk were even underfed by 11%. All studied trace minerals were fed in excess for the majority of cows. Cobalt was fed on average 480% above requirements regardless of the stage of lactation. For Cu, Fe, Mn, and Zn, rations for cows below 21 days in milk were fed 23% (95% confidence interval: 15–32), 930% (849–1019), 281% (251–314), and 35% (22–47) above the recommendations, respectively, and were closer to the requirements than after 21 days in milk. These results show that most nutritionists are aware that precision feeding regarding P is important to minimize detrimental environmental impacts of dairy production. However, some efforts should be made to limit trace mineral overfeeding to ensure environmental resiliency.

## 1. Introduction

It is well accepted that many minerals are required to support vital functions in mammals [1]. For instance, phosphorus (P) is a component of cell membranes and contents and has a role in energy metabolism, whereas copper (Cu) and zinc (Zn) are involved in immune function, have antioxidant properties, and are cofactors of numerous enzymes [2]. As mineral supplies from forages might not be sufficient to meet requirements of high-producing lactating cows and as their bioavailability in natural feedstuffs may be relatively low [1], it is then a common practice to add commercial mineral supplements to the diet, sometimes combined with commercial energy or protein supplements. Fulfilling mineral requirements offers several challenges to cow nutritionists. As trace mineral concentrations of homegrown farm ingredients are not commonly analyzed by feed laboratories, due to the fact that the near infrared method for their prediction is not reliable [3], some nutritionists either use mineral concentrations from available references in the formulation software or only consider the commercial mineral supplement as a known source of trace minerals for the animals when formulating rations. Nevertheless, it is possible that the software references used to formulate rations are not really representative of the ingredients actually fed to the cows, as mineral concentrations of feed change according to the soil types and regions where the crop grew [4]. This could lead to either the under- or over-supplying of minerals. On the other hand, not considering homegrown ingredients as a source of trace minerals in the diet could be a cause of overfeeding those minerals. In addition to these challenges, mineral absorption data of ingredients are extremely limited, and complex interactions with minerals could occur within the rumen [1]. For instance, it has been reported that Cu absorption is compromised of high dietary molybdenum and sulfur. 

Underfeeding minerals could have detrimental effects on animal productivity and health [5], whereas overfeeding them could have negative effects on the environment and cow health. A review from Goff [6] discussed the contradictory effect of minerals such as Cu, Fe, manganese (Mn), and Zn as pro-oxidants and antioxidants. They have the ability to obtain electrons from other molecules and play a role of antioxidant, but, if supplied in excess, they could cause tissue damage by increasing free ionized metal concentrations. As absorption of most minerals in ruminants is low, a large proportion of ingested minerals is found in manure [7,8]. In some cases, this is exacerbated by a homeostatic control in which there is a decrease in mineral absorption efficiency as the dietary mineral increases [9,10]. Hence, if manure with high mineral concentrations is spread on the field, after many years of manure application, this could cause mineral build up in the soil and potentially decrease the crop yield if the mineral maximal tolerable limit is reached and cause water pollution by mineral leaching or runoff [11,12]. In early lactation, supplying organic trace minerals above the National Research Council (NRC) [1] recommendations had positive effects on the immune system and decreased inflammation and oxidative stress [13,14], whereas no significant effect was noted in mid-lactation cows [15].

A survey conducted in Wisconsin herds has shown that 50% and 94% of herds fed rations exceeding Zn and Cu requirements of the NRC, respectively [16]. In a survey conducted in 50 UK herds, Sinclair and Atkins [17] found that rations fed to cows were in excess of Cu, Mn, and Zn requirements. Similar results were obtained by Castillo, et al. [18] in Californian herds. These authors nevertheless mentioned that P was the mineral fed closest to the requirement [18]. Challenges faced by dairy nutritionists to address the mineral requirements mentioned above could explain why many of them formulate rations exceeding requirements, which could be considered as an insurance to fulfill cow needs. Moreover, it should be noted that most rations are formulated on the basis of a group of animals and a cow-to-cow variation in mineral requirements are expected. In order to account for this variability within a group, modest mineral overfeeding relative to the average requirements of the group is the current practice. Nevertheless, this raises the concern that the current situation is far from being precision feeding regarding minerals. To achieve this, rations should be formulated to fulfill cow requirements to optimize productivity and health while mitigating environmental impacts of high mineral excretion in manure. The objective of this study was to obtain accurate information on P and 5 trace minerals, i.e., cobalt (Co), Cu, Fe, Mn, and Zn concentrations in diets of commercial dairy herds in the province of Québec, Canada and to compare those concentrations with dietary recommendations from three different sources (NRC, the Institut National de la Recherche Agronomique (INRA), and the European Federation of Animal Science (EAAP)). In contrast to trace minerals, the impact of excess dietary P has been widely studied as a major ecosystem pollutant [19,20] and the precision P feeding concept has been reported [21,22]. Hence, P is included in this manuscript as a point of comparison between the current feeding management in commercial dairy herds for a mineral for which the environmental effect of overfeeding has been well studied and for those for which it has been less studied (Co, Cu, Fe, Mn, and Zn).

## 2. Materials and Methods

All procedures of this study were approved by the Animal Care committee from Université Laval, Québec, Canada following the guidelines of the Canadian Council on Animal Care [23].

### 2.1. Herds and Management

This experiment was primarily conducted to evaluate the impact of diet management and composition on vitamin B_12_ concentration in milk, to evaluate the Canadian dairy herd improvement model and to assess the nitrogen efficiency of eastern Canadian dairy herds [24,25,26]. Thus, herds and management have been previously described in those papers. Briefly, 100 eastern Canadian commercial dairy herds, all located in the province of Québec, were visited from October 2014 to June 2015. To participate in the cross-sectional study, dairy farms had to have Holstein cows as the main breed, to milk cows twice a day, to record milk yield and milk composition during 2 consecutive milkings through the dairy herd improvement agency (Lactanet, formerly Valacta, Canadian Network for Dairy Excellence, Sainte-Anne-de-Bellevue, QC, Canada), and to feed cows according to well-recognized recommendations such as the National Research Council [1] or the Cornell Net Carbohydrate and Protein System. Producers having a herd that fulfilled the previous requirements were contacted by phone and the participation was on a voluntary basis. Herd size ranged from 16 to 113 lactating cows who were housed either in tie-stall (*n* = 98) or free-stall (*n* = 2) barns. The morning milking interval varied from 10.7 to 14.8 h. Total mixed ration (TMR) was used by 31 herds, automatic component feeding system (AFS) by 49, and manual component feeding system (MCF) by 20. 

### 2.2. Data Collection

Herd visits were conducted during 3 consecutive milkings. At the first visit, which was during the morning milking, all ingredients were sampled as well as the quantities of each ingredient given were recorded, as previously described [25]. In summary, for TMR herds, quantities of each ingredient offered to the cow were directly recorded from the scale of the mixing system. A validation was performed by weighing TMR quantities given to 10 cows. Regarding AFS and MCF herds, when forage, such as chopped grass or corn silage, was supplied using a cart, the quantities of forage fed to each feeding group were weighed for 10 cows, whereas, for hay or silage bales, they were weighed with an electronic scale (OCSB3 Compact Crane Scale; Anyload Transducer Co. Ltd., Burnaby, BC, Canada). For component feeds in AFS herds, the quantities of ingredients for each individual were obtained from the computer of the feeding robot, which were first calibrated by comparing the predicted and actual weights of each ingredient. Lastly, concerning MCF herds, the quantities of each component were weighed before offering it to the cow. Feed samples were stored at −20 °C until analysis. At the second and third milkings, respectively, an evening and a morning milking, visits were scheduled to occur simultaneously with a regular dairy herd improvement test. Milk yield was recorded, and milk samples preserved with bronopol were taken using calibrated in-line milk meters at each milking. Milk samples were immediately sent to the Lactanet laboratory for milk component analysis. The estimated body weight (BW) of cows were obtained by measuring the hearth girth circumference and applying the equation of Yan et al. [27]. Data on individual cows, such as parity, days in milk (DIM), number of days pregnant at the time of the visits, were obtained from the Lactanet database. 

### 2.3. Analyses

Feed samples were thawed, then placed in an air-forced oven at 55 °C for 48 h for dry matter (DM) determination and ground at 1 mm. Samples were sent to a commercial laboratory (SGS Canada, Guelph, ON, Canada) for analysis by wet chemistry for crude protein (method 990.03; AOAC International [28]), ADF (Ankom Technology Method 12; solutions as in method 973.18; AOAC International [28]), aNDF (Ankom Technology Method 13; solutions as in Van Soest et al. [29] with the inclusion of heat-stable α-amylase), crude fat (Ankom Technology Method 2; AOCS [30]), starch (method 996.11; AOAC International [28]), and mineral profile (inductively coupled plasma; methods 985.01 and 965.09; AOAC International [28]). Net energy of lactation and nonfiber carbohydrates were calculated according to the NRC [1] equations. For the purposes of the other studies conducted on the same dataset [24,25], not all individual ingredient samples were sent to the laboratory. Indeed, using the DM percentage of each ingredient of a ration, weighted TMR samples were reconstituted and sent to the laboratory for analysis. Moreover, for herds not using TMR management, a weighted pool, on a DM basis, of all silages offered per feeding group were analyzed. Milk samples from the evening and morning milking were separately analyzed for fat, protein, and lactose concentrations by mid-infrared reflectance spectrometry (MilkoScan FT 6000, Foss, Hillerød, Denmark) at Lactanet laboratory.

### 2.4. Calculations

Feed ingredients were divided into 9 distinct categories: (1) Forage; (2) Corn grain; (3) Other cereals; (4) Energy commercial supplements; (5) Fat supplement; (6) Soy products; (7) Protein commercial supplements; (8) Minerals and vitamins; (9) Feed additives. To be considered as energy or protein commercial supplements, feeds had to have at least 30% of non-fiber carbohydrates and 30% of crude protein on a DM basis, respectively. Other cereals included oat, barley, wheat, and mixed cereals, whereas feed additives included yeast, antitoxin, and sodium bicarbonate. The percentages of each category listed above in the diet were computed by dividing the daily quantity given of each ingredient or group of ingredients included in the category by the total ration offered on a DM basis using Proc SQL of SAS, version 9.4 [31]. For AFS and MCF herds, the nutrient composition of rations was computed by multiplying the percentage of each category on a DM basis by its nutrient composition obtained by wet chemistry, whereas the diet nutrient composition of TMR herds was directly obtained from the wet chemistry analysis of the weighted TMR samples. It was then possible, for AFS and MCF herds only, to obtain the percentage of contribution of dietary P, Co, Cu, Fe, Mn, and Zn concentrations from each ingredient category.

Dietary requirements of P, Co, Cu, Fe, Mn, and Zn were calculated per cow on the basis of 3 references, i.e., NRC [1], INRA [32], and EAAP [33]. The factorial approach was used regarding the NRC recommendations, except for Co, for which there was one recommendation regardless of physiologic stages. Regarding INRA and EAAP recommendations, the factorial approach was only used for P. All equations to obtain dietary requirements are presented in Supplementary Table S1. The factorial approach was based on requirements for maintenance, lactation, pregnancy, and growth, whenever applicable. Except for Cu, for which there was a pregnancy requirement throughout the gestation, the pregnancy requirement was only considered during the last third of the gestation. Growth requirement was only considered for primiparous cows. For requirement equation purposes, some calculations needed to be made as follows: (1) fat-corrected milk for lactation requirements = (0.4 × daily milk yield) + (15 × (milk fat concentration/100) × daily milk yield); (2) DM intake for P maintenance requirements calculated as per NRC [1], i.e., DM intake (kg/d) = (0.372 × fat corrected milk + 0.0968 × BW ^0.75^) × (1 − ℮^(−0.192 × (week of lactation + 3.67))^); (3) within each herd, average daily gain during the first lactation for primiparous growth requirements = (herd average BW of second parity cows—herd average BW of primiparous cows)/herd average calving interval; (4) mature BW for primiparous P growth requirements = herd average BW of third and more parity cows; and (5) number of days of pregnancy = DIM at the time of sampling—(lactation plus dry period length—282 d of gestation), when (lactation and dry period length—282 d of gestation) was lower than 55 DIM, the cow was considered as non pregnant at the time of the visit and it was hypothesized that the reason for the end of the lactation cycle was due to culling instead of calving. For P, Cu, Fe, Mn, and Zn requirements from NRC, and P requirements from INRA and EAAP, they were first obtained as absorbed mineral requirements and an absorption coefficient was applied to obtain the daily dietary recommendations. According to the NRC models [1], absorption coefficients of Cu is 0.04, 0.1 for Fe, 0.0075 for Mn, 0.15 for Zn, 0.64 for concentrate ingredients, and 0.7 for forage regarding P. Hence, average herd concentrates and forage percentages were calculated, and these last absorption coefficients were applied for P. The same absorption P coefficients were used for INRA recommendations, whereas a coefficient of 0.7 was used for all ingredients for EAAP recommendations. Daily dietary recommendations were divided by the predicted DM intake based on the NRC calculation to obtain the dietary recommendations as the percent of P and mg/kg DM of other minerals. The percentage of under or overfeeding minerals relative to the recommendations was calculated as the mineral concentrations provided by the diet minus the dietary recommendation and then divided by the dietary recommendation. Hence, from this calculation, negative values indicate underfeeding, whereas positive values imply overfeeding. The percentages of mineral NRC recommendations fulfilled by individual feed categories were calculated as the mineral concentrations of the feed category divided by the mineral requirements. 

### 2.5. Statistical Analyses

Descriptive statistics were obtained with Proc UNIVARIATE of SAS. As for some herds, especially TMR herds, cows within the same feeding group, often based on DIM, theoretically received the same amount of minerals, it was decided to group cows according to DIM within each herd as follows: (1) at or below 21 DIM; (2) between 22 and 80 DIM; (3) between 81 and 199 DIM and; (4) at or above 200 DIM. These thresholds respectively represent fresh, early-, mid-, and late-lactation periods. Hence, in each herd, there were 4 averaged mineral dietary concentrations, one per DIM category. The DIM category can be considered as the experimental unit. Proc MIXED of SAS was used to assess the impact of the DIM category as the fixed effect on the characteristics of each group, on the dietary recommendations of mineral when a factorial approach is used, on the dietary mineral concentrations, and on the percentage of under or overfeeding minerals relative to the recommendations. A Tukey’s honestly significant difference test was performed when results reached significance or a tendency. Normality was visually assessed with residual plots. This condition was violated regarding dietary concentrations of Co, Cu, Fe, Mn, percentages of dietary Cu, Fe, Mn concentrations relative to the recommendations, and then log transformation was chosen to overcome this issue. Geometric means and a 95% confidence interval from back-transformed data were presented for these variables when indicated in Tables. Significance was declared at *p* ≤ 0.05.

## 3. Results and Discussion

### 3.1. Descriptive Statistics between Feeding Systems

A total of 4430 Holstein cows were involved in that cross-sectional study (TMR, *n* = 1757; AFS, *n* = 2014; and MCF, *n* = 659). Table 1 shows descriptive statistics regarding cow characteristics and diet composition according to the three feeding systems. As suggested by the number of participating cows per herd, it could be observed that MCF management was associated with smaller herds compared with AFS and TMR. An average cow had 181 DIM, 2.5 lactations, and weighed about 678 kg. Daily milk yield averaged 31.7 kg with fat and protein concentrations of 4.13% and 3.35%, respectively. A typical diet contained 67.7% of forage and 32.3% of concentrate with 15.2% of crude protein, 1.57 Mcal/kg of NE_L_, and 37.6% of aNDF on a DM basis. The predicted DMI averaged 23.2 kg/d. 

Regarding our studied minerals, i.e., P, Co, Cu, Fe, Mn, and Zn, Table 1 shows the wide variation of mineral feeding management among herds in this cross-sectional trial. The least variation between centiles 1 and 99 was obtained for dietary P concentration.

### 3.2. Mineral from Feed Ingredient Categories

Table 2 indicates mineral concentrations of feeds by categories. Percentages of NRC requirements of selected minerals fulfilled by each feed category are depicted in Table 3. It was not possible from the current dataset to discriminate mineral concentrations of individual forages. It is interesting to note the wide variability of mineral requirements satisfied by ingredients and mineral concentrations of ingredients. Indeed, P requirements fulfilled by the forage in the ration varied from 32.7% (percentile 1) to 93.0% (percentile 99), whereas Co requirement fulfillment by forages ranged from 0.0% (percentile 1) to 948.2% (percentile 99). These results highlight the fact that mineral concentrations of ingredients are affected by several factors such as soil type [4], soil contamination, and sampling method. This could also be the case for homegrown components such as corn grain, other cereals, and soy products. Disregarding mineral interactions that could occur in the rumen and taking into account absorption coefficients, results suggest that, for more than 50% of the participating herds, forages were sufficient to attain the NRC Co, Fe, and Mn requirements. Nevertheless, forage source solely did not suffice to reach the requirement adequacy of P, Cu, and Zn (Table 3). Sprinkle et al. [4] also obtained similar results. Nevertheless, very little data are available on the mineral absorption efficiency of basal diet ingredients, which could have a major effect on supply calculations. The huge variability in percentages of mineral requirements fulfilled by commercial energy and protein supplements and minerals and vitamins could be explained by the fact that, in some herds, minerals and vitamins were added in the commercial energy or protein supplement. Hence, in some rations of these herds, no additional mineral and vitamin supplement was added, as observed in Table 1. In 50% of herds, the mineral supplement alone fulfilled the daily requirements, or was close to these, for Co, Fe, and Mn. It is interesting to note that the mineral supplement satisfied from 0.9% to 47.9% (percentile 1 to 99) of the P requirement, hence not exceeding 100% of needs. This shows that nutritionists pay special attention to this mineral in order to avoid an overall diet excess of P. Median concentrations of Cu and Zn of mineral and vitamin supplements obtained in the current study were similar to what has been reported by Li et al. [16]. 

### 3.3. Recommendations According to DIM

Averaged DIM were 12.5, 50.9, 140.7, and 291.3 ± 1.7 by DIM category ≤21, between 22 and 80, between 81 and 199, and ≥200, respectively (*p* < 0.0001; Table 4). As expected, the milk yield was greater and milk fat and protein concentrations were lower during the lactation peak between 22 and 80 DIM than other DIM categories. Except for Co, for which a non-factorial approach was used to compute requirements, studied mineral recommendations from NRC were greater in the fresh group (<21 DIM) compared with other DIM categories (*p* < 0.0001; Table 4). The same results were obtained for P recommendations from INRA and EAAP. This could partly be explained by lower predicted DMI in those cows. Moreover, those cows had greater fat-corrected milk than cows in mid- and late-lactation, and this implies that they have higher requirements to support lactation. Greater Cu, Fe, Mn, and Zn requirements above 200 DIM compared with between 81 and 199 DIM could be explained by the increased demand for pregnancy. There is no specific recommendation for Fe in INRA tables and a non-factorial approach regarding Co, Cu, Mn, and Zn was adopted by the committee [32]. A non-factorial approach was also used for these last minerals by the EAAP committee as well as for Fe [33]. As also outlined by Sinclair and Atkins [17], requirement dissimilarities exist between recommendation sources. This is the case regarding Mn, where NRC recommendation is well below INRA and EAAP recommendations. It is worth noting that Weiss and Socha [34] have found that Mn NRC requirements might be underestimated.

Except for Co and Fe, dietary concentrations of P, Cu, Mn, and Zn changed with DIM categories (*p* < 0.02; Table 5), where they were greater <21 DIM than in late lactation. This is in line with NRC recommendations that support the increasing demand for milk production coupled with the limited DMI during this period. Regarding NRC recommendations for P, Cu, Fe, Mn, and Zn, dietary concentrations were closer to the requirements before 21 DIM than thereafter in the lactation (*p* < 0.0001; Table 5). However, as a non-factorial approach was used for studied trace minerals in INRA and EAAP recommendations and as dietary concentrations were greater in early postpartum, trace mineral overfeeding in early lactation was higher than in late lactation. It could be noted that for all trace minerals, regardless of the recommendation sources and DIM categories, the average dietary concentration exceeded the guidelines, as previously observed [16,17,18]. None of these, however, surpassed the maximum tolerable levels for Co, Mn, Fe, and Zn [35]. Mineral toxicity in animals is quite unusual, as an adaptation mechanism occurs to increase manure excretion according to the increase in supply [1]. In a review, López-Alonso [5] have stressed that this current practice of providing more minerals than needed in intensive systems could have detrimental effects on ecosystems. 

### 3.4. Phosphorus

Average dietary P concentrations among DIM categories ranged from 0.37% to 0.40% of DM. Among the studied minerals, dietary P was found to be the closest to the requirements regardless of the recommendation sources (Table 5). This was also observed by Castillo et al. [18]. Phosphorus is among the most studied mineral in regard to its environmental impact. It is well recognized that overfeeding P leads to increased P excretion in manure which in turn augments the risk of P eutrophication of waterbodies and algal bloom when manure is spread on fields [36]. Agriculture explains a major part of P accumulation in water bodies [20,21]. Hence, this is why, in many countries, the amount of P that could be applied to the land is regulated by laws [1]. This is the case in the province of Québec, Canada. This regulation has been accompanied with a decrease of dietary P for the past years [37], followed by a subsequent reduction of P excretion in manure [7]. Even though P was fed closer to the recommendations, a wide range of percentage relative to the recommendations could be observed among the 100 herds (Table 6). Indeed, it ranged from −34% to 64% according to NRC recommendation calculations. Rather than causing an environmental threat with overfeeding, P underfeeding could have a detrimental effect on cow health [1].

### 3.5. Trace Minerals

In the last years, studies have been conducted to evaluate the effect of trace mineral supplementation sources, i.e., either inorganic or organic sources, especially in early lactation, on cow performance, immunity, health, and oxidative metabolism [13,14,38,39]. Unfortunately, it is not possible from the current assessment to discriminate the source of trace mineral supplement given to the cows. Milk production per cow has increased remarkably over the last years and whether trace metal requirements as per NRC [1] is sufficient to express optimal performance and metabolism function has been questioned [40]. Hence, some studies have investigated the effect of feeding greater trace-metal concentrations than the NRC recommendations [14,15]. Regardless of the trace-metal sources, dietary Co, Cu, Mn, and Zn concentrations in the study of Osorio et al. [14] represented percentiles 90, 10, 35, and 38, respectively, of the current diet distribution of the 100 herds for cows below 21 DIM. This means that, although dietary concentrations of Co, Cu, Mn, and Zn were already higher than NRC recommendations in Osorio et al. [14], 10%, 90%, 65%, and 62% of herds in the current study provided even greater amounts to fresh lactating cows. 

Cows do not have a Co requirement per se, but the microorganisms dwelling in their rumen do need Co to synthesize vitamin B_12_ [41], which, in turn, is needed by the cow. Co was fed in excess between 452% and 509%, 102% and 123%, and 507% and 569% relative to the NRC, INRA, and EAAP recommendations, respectively, among DIM categories (Table 5). Moreover, Table 6 shows that all herds, if following NRC and EAAP recommendations, fed dairy cows with an excess of Co. In a cross-sectional study involving American and Canadian farms, Duplessis et al. [42] also found that Co concentrations exceeded the NRC requirement and hence they failed to find a relationship between dietary Co concentration and plasma vitamin B_12_ concentration. Co is usually not reported in surveys assessing the difference between dietary mineral concentrations and the requirements. 

Dietary Cu concentration was greater in the ≤21 than in the ≥200 DIM category (*p* = 0.02; Table 5) and was similar to what has been reported in Wisconsin and California herds [16,18]. Bidewell et al. [43] reported a case of Cu poisoning for cows receiving a ration having 50 mg/kg DM of Cu. The maximum tolerable level of Cu was set at 40 mg/kg of DM [35]. One herd was fed a ration with Cu concentration above 40 mg/kg DM in the current study. Copper is the trace mineral having the greatest potential to cause toxicity, as the difference between the requirement and the toxic level is small [6]. Hence, nutritionists should pay special attention to Cu to avoid overfeeding. The median of the percentage of dietary Cu concentration in excess to the NRC recommendation was 52% and was the closest to the NRC recommendation regarding trace minerals (Table 6). Dietary Cu absorption is known to decrease with increasing dietary sulfur and molybdenum [1]. Unfortunately, molybdenum concentration in the diet was not available in the current study. Nevertheless, results suggested that dietary sulfur averaged 0.21% of DM (Table 1), which is close to the required sulfur concentration [1]. 

Dietary Fe concentration did not change according to DIM categories (*p* = 0.17) and averaged 226 (SD: 88) mg/kg of DM (Table 5). Regarding NRC recommendations, as lactation progressed, the percentage of dietary Fe concentration relative to the recommendation progressively increased (*p* < 0.0001). No significant effect of DIM categories was observed for the Fe EAAP recommendation regarding the percentage of dietary concentration over the requirements (*p* = 0.17). Among the studied minerals, Fe was the most overfed according to the NRC and EAAP recommendations (Table 5), as also previously observed [17,18]. Castillo et al. [18] explained this result by the fact that forages contain large amounts of Fe, but with low bioavailability, lowering the risk of toxicity for the animal [35]. Moreover, Fe is rarely intentionally added in the mineral supplement. In the current study, for both NRC and EAAP recommendations, all cows were fed above the requirements as percentile 1 was 469 and 63% relative to the recommendations, respectively (Table 6). 

The dietary concentration of Mn was greater below 21 than above 81 DIM (*p* = 0.02; Table 5). The dietary recommendation of Mn was greater for INRA and EAAP than NRC (Table 4). This is why the Mn concentration in the diet was closer to the INRA and EAAP than the NRC recommendations (Table 5). As mentioned above, Weiss and Socha [34] have found that Mn requirements for lactation cows are about 1.6 higher than the NRC recommendation. As for other trace minerals, Mn was also fed in excess, as also observed by others [17,18], and it was different according to the stage of lactation and recommendation sources (*p* ≤ 0.02; Table 5). According to the INRA and EAAP recommendations, some cows were fed below their requirements (Table 6). Nevertheless, this was not the case according to the NRC recommendations, as the dietary Mn concentration was 107% above the requirements at percentile 1. Manganese toxicity is not a common problem in ruminant, as the maximum tolerable amount is 2000 mg/kg of DM [1] and no adverse signs were observed when dietary Mn was below this threshold [35]. In the current study, the highest Mn concentration in the diet reached 285 mg/kg of DM. 

The dietary Zn concentration was greater in cows below 21 than above 81 DIM (*p* = 0.001; Table 5). Zinc concentrations in diets observed by Li et al. [16] in Wisconsin, USA were similar to the current assessment. Nevertheless, surveys conducted in European and in central and northern England dairy farms [17,44] have shown that Zn concentrations in the diet were smaller by between 14% and 30% than in the current study, probably caused by the European Union legislation regarding trace minerals [45]. Cows below 21 DIM were fed closer to their Zn NRC recommendations than other DIM categories (*p* < 0.0001), whereas the opposite was obtained regarding INRA and EAAP requirements (Table 5). About 90% of cows were fed above their Zn requirements, regardless of the source. Along with Cu, Zn is one of the trace minerals fed closest to the NRC requirements (Table 6), with a median of 65% in excess of the requirements. Sobhanirad et al. [46] and Sobhanirad and Naserian [47] did not find adverse effects of feeding rations with Zn concentration greater than 500 mg/kg of DM. In the current study, all cows were fed below the Zn concentration used in Sobhanirad et al. [46].

### 3.6. Study Limitations

As conducted, the study has some limitations that should be taken into account while interpreting results. For instance, a single sample of each ingredient has been taken for TM analysis in each farm. This assumes that samples were representative of what cows had received the day of the visit. This study also relies on the accuracy of mineral analyses of feed ingredients. In a previous study [48], authors sometimes obtained major differences in Co concentration of feed ingredients between two laboratories using different machines. It should also be noted that the current analysis used a calculation to predict DM intake for P maintenance requirements as actual DM intake was not recorded. This is the same for predicted BW calculated using heart girth circumference. Moreover, some results rely on the accuracy of mineral absorption coefficients found in the literature. These characteristics could have led to result uncertainty. 

## 4. Conclusions

Regarding P, Cu, Mn, and Zn dietary concentrations per kg of DM, these decreased as lactation progressed. Among the studied minerals, P was the one closest to the precision feeding concept, especially in early lactation. Regarding other selected minerals, above 75% of cows received a ration with excess Co, Cu, Fe, Mn, and Zn. In addition, Fe and Co were among the most overfed minerals, regardless of lactation stage for Co. One herd was fed a dietary Cu concentration above the maximum tolerable level, and this should be avoided to prevent toxicities. Except for commercial products with mineral addition, forage was the major source of minerals, when disregarding ruminal interaction that could occur and mineral absorption efficacy. Forages were sufficient to fulfill the NRC Co, Fe, and Mn requirements for more than 50% of the participating herds. This paper shows that efforts should be made in commercial dairy herds in Québec, Canada regarding precision feeding of trace minerals. This is of major importance for animal health and also to ensure ecosystem resiliency and sustainability.

## Figures and Tables

**Table 1:** Descriptive statistics regarding cow and diet characteristics across feeding systems.

Items	Total Mixed Ration			Automatic Component Feeding			Manual Component Feeding		
	Average (SD)	Centile 1	Centile 99	Average (SD)	Centile 1	Centile 99	Average (SD)	Centile 1	Centile 99
Cows per herd	56.7 (19.2)	23	110	40.6 (12.1)	23	80	32.1 (19.7)	17	109
Cow characteristics									
Days in milk	186 (117)	8	513	178 (113)	7	489	174 (110)	9	454
Parity	2.4 (1.5)	1	7	2.5 (1.6)	1	7	2.6 (1.6)	1	7
Estimated body weight (kg)	683 (60)	542	829	673 (56)	548	803	676 (58)	553	820
Milk yield (kg/day)	32.4 (9.7)	10.2	56.4	31.6 (9.5)	10.2	53.4	29.8 (8.6)	10.6	51.9
Milk fat (%)	4.15 (0.63)	2.84	6.02	4.12 (0.61)	2.88	5.86	4.09 (0.58)	2.83	5.59
Milk protein (%)	3.37 (0.37)	2.64	4.36	3.33 (0.35)	2.63	4.30	3.35 (0.36)	2.64	4.25
Milk lactose (%)	4.57 (0.19)	3.97	4.95	4.57 (0.19)	3.96	4.94	4.56 (0.19)	4.00	4.92
Predicted DMI ^1^ (kg)	23.7 (3.5)	13.6	32.0	23.1 (3.4)	14.0	30.7	22.5 (3.1)	14.3	29.5
Diet characteristics									
Ingredients (% of DM)									
Forage ^2^	65.2 (9.3)	47.4	84.9	68.0 (7.8)	54.1	91.1	70.6 (9.5)	47.1	89.0
Corn grain	17.7 (9.1)	0.0	40.9	11.2 (9.8)	0.0	33.0	9.1 (12.7)	0.0	40.2
Other cereals ^3^	1.0 (2.8)	0.0	12.5	5.6 (9.1)	0.0	33.6	2.6 (6.7)	0.0	28.1
Energy commercial supplement ^4^	2.6 (6.1)	0.0	34.1	7.5 (12.7)	0.0	40.5	12.6 (11.4)	0.0	35.2
Fat supplement	0.4 (0.7)	0.0	3.6	0.1 (0.3)	0.0	1.5	0.0 (0.0)	0.0	0.0
Soy products	4.8 (4.8)	0.0	14.7	1.4 (2.7)	0.0	11.5	0.6 (1.8)	0.0	8.5
Protein commercial supplement ^5^	5.2 (4.6)	0.0	20.1	5.2 (6.6)	0.0	28.2	3.7 (6.5)	0.0	31.0
Minerals and vitamins ^6^	0.9 (0.8)	0.0	2.5	0.7 (0.7)	0.0	3.4	0.7 (1.0)	0.0	3.4
Total concentrate	34.8 (9.3)	15.1	52.6	31.9 (7.8)	8.9	46.0	29.5 (9.5)	11.0	52.9
Nutrient composition (% of DM, unless otherwise specified)									
Crude protein	15.2 (1.1)	13.0	19.1	15.6 (1.6)	12.3	21.2	14.2 (1.8)	11.6	19.2
Net energy of lactation (Mcal/kg of DM)	1.55 (0.05)	1.42	1.65	1.59 (0.06)	1.44	1.72	1.53 (0.09)	1.31	1.72
Starch	19.4 (6.0)	1.1	27.9	17.1 (5.2)	5.6	31.9	16.0 (5.6)	5.2	28.2
Acid detergent fiber	21.0 (3.1)	15.3	29.2	21.9 (2.9)	15.2	30.0	24.0 (3.8)	14.7	30.7
Neutral detergent fiber	36.2 (5.3)	25.5	49.1	37.2 (4.7)	27.8	49.6	40.9 (4.7)	29.1	52.3
Lignin	2.6 (0.9)	0.8	5.6	3.7 (1.1)	1.5	6.5	3.8 (1.0)	1.8	5.7
Non-fiber carbohydrate	42.4 (4.7)	29.1	50.4	40.0 (4.1)	28.1	48.6	38.4 (4.5)	28.3	48.5
Fat	3.2 (0.7)	2.3	5.0	3.3 (0.7)	2.1	5.6	2.9 (0.7)	1.5	4.5
Ash	7.0 (0.9)	5.4	10.2	7.8 (1.4)	4.9	12.2	7.7 (1.1)	6.2	10.1
Ca	0.78 (0.18)	0.38	1.23	0.90 (0.24)	0.51	2.03	0.79 (0.19)	0.50	1.19
P	0.37 (0.05)	0.23	0.48	0.39 (0.06)	0.27	0.60	0.37 (0.07)	0.26	0.51
K	1.56 (0.38)	0.75	2.92	1.69 (0.32)	1.00	2.49	1.82 (0.32)	1.04	2.62
Mg	0.28 (0.04)	0.20	0.36	0.29 (0.06)	0.18	0.50	0.28 (0.05)	0.21	0.39
S	0.22 (0.04)	0.15	0.30	0.21 (0.03)	0.16	0.35	0.20 (0.05)	0.13	0.33
Co (mg/kg of DM)	0.60 (0.24)	0.30	1.50	0.60 (0.26)	0.13	1.24	0.66 (0.30)	0.29	1.57
Cu (mg/kg of DM)	17 (5)	10	34	18 (6)	9	44	16 (7)	7	32
Fe (mg/kg of DM)	248 (77)	81	407	228 (95)	96	525	162 (54)	95	296
Mn (mg/kg of DM)	65 (18)	27	123	77 (32)	33	191	62 (27)	31	136
Zn (mg/kg of DM)	76 (21)	33	144	88 (46)	40	353	72 (34)	26	149

^1^ Predicted dry matter intake using equations from National Research Council [1]. ^2^ Forage category included mixed grass and legume silage, corn silage, and mixed hay. ^3^ Oat, barley, wheat, and mixed cereals included. ^4^ Defined as non-fiber carbohydrates > 30% of DM. ^5^ Defined as CP > 30% of DM. ^6^ Commercial blend. For some herds, vitamins and minerals were included in the commercial energy or protein supplements. Abbreviations: DM = dry matter; DMI = dry matter intake; SD = standard deviation.

**Table 2:** Selected mineral concentrations of feed-category ingredients ^1^.

Item	Median	Centile 1	Centile 25	Centile 75	Centile 99
Forage ^2^					
P (% DM)	0.27	0.17	0.24	0.32	0.40
Co (mg/kg DM)	0.31	0.00	0.21	0.50	1.36
Cu (mg/kg DM)	8.9	5.0	7.4	10.0	18.7
Fe (mg/kg DM)	131	48	87	215	630
Mn (mg/kg DM)	33	13	24	48	109
Zn (mg/kg DM)	28	16	24	32	62
Corn grain					
P (% DM)	0.30	0.16	0.27	0.32	0.44
Co (mg/kg DM)	0.21	0.00	0.11	0.34	0.83
Cu (mg/kg DM)	2.5	1.1	1.6	3.1	15.7
Fe (mg/kg DM)	31	20	26	35	148
Mn (mg/kg DM)	5	3	4	6	61
Zn (mg/kg DM)	21	6	19	25	62
Other cereals ^3^					
P (% DM)	0.40	0.31	0.36	0.44	0.51
Co (mg/kg DM)	0.31	0.00	0.21	0.43	0.63
Cu (mg/kg DM)	6.7	2.0	5.8	8.4	15.7
Fe (mg/kg DM)	60	34	50	82	164
Mn (mg/kg DM)	18	6	15	35	61
Zn (mg/kg DM)	40	23	36	49	62
Energy commercial supplement ^4^					
P (% DM)	0.62	0.30	0.54	0.77	1.19
Co (mg/kg DM)	0.99	0.11	0.91	1.79	4.98
Cu (mg/kg DM)	32.0	9.1	10.2	51.9	118.0
Fe (mg/kg DM)	290	139	203	611	641
Mn (mg/kg DM)	132	12	66	237	562
Zn (mg/kg DM)	173	23	56	279	526
Soy products ^5^					
P (% DM)	0.74	0.51	0.74	0.74	0.80
Co (mg/kg DM)	0.31	0.21	0.31	0.31	0.52
Cu (mg/kg DM)	14.0	8.5	14.0	14.0	18.6
Fe (mg/kg DM)	128	90	128	128	154
Mn (mg/kg DM)	36	22	36	36	36
Zn (mg/kg DM)	53	48	53	53	66
Protein commercial supplement ^6^					
P (% DM)	0.86	0.36	0.76	1.22	1.81
Co (mg/kg DM)	2.26	0.00	1.36	2.70	5.69
Cu (mg/kg DM)	74.3	9.5	48.8	94.8	163.8
Fe (mg/kg DM)	442	129	310	622	1177
Mn (mg/kg DM)	288	27	170	326	686
Zn (mg/kg DM)	355	43	262	466	1341
Minerals and vitamins ^7^					
P (% DM)	4.43	0.00	3.51	7.11	18.70
Co (mg/kg DM)	13.00	0.30	11.00	16.50	29.00
Cu (mg/kg DM)	567.3	0.0	505.0	709.2	1280.5
Fe (mg/kg DM)	3087	21	2772	4690	12031
Mn (mg/kg DM)	2002	37	1882	2489	5215
Zn (mg/kg DM)	2797	62	2537	3189	14531

^1^ Herds using a total mixed ration were not included in this analysis, due to missing information. Herds not including a feed category in their ration were excluded from the analysis of this given feed category. ^2^ Forage category included mixed grass and legume silage, corn silage, and mixed hay. ^3^ Oat, barley, wheat, and mixed cereals included. ^4^ Defined as non-fiber carbohydrates >30% of DM. ^5^ Only 4 different samples were analyzed in this category. ^6^ Defined as CP >30% of DM. ^7^ Commercial blend. For some herds, vitamins and minerals were included in the commercial energy or protein supplements. Abbreviations: DM = dry matter.

**Table 3:** Percentages of mineral recommendations fulfilled by individual feed categories for the 100 dairy herds according to the National Research Council ^1^.

Item (%)	Median	Centile 1	Centile 25	Centile 75	Centile 99
Forage ^2^					
P	55.2	32.7	47.5	63.7	93.0
Co	196.2	0.0	127.3	288.0	948.2
Cu	53.7	31.4	46.2	63.4	97.8
Fe	775.7	219.1	550.3	1135.5	3484.2
Mn	181.2	73.4	130.1	259.5	469.2
Zn	43.7	22.8	36.4	53.1	95.5
Corn grain					
P	14.1	2.9	9.2	19.5	37.4
Co	22.5	0.0	7.8	44.6	139.1
Cu	3.4	0.4	1.9	5.3	22.2
Fe	33.3	5.1	21.4	58.4	221.0
Mn	6.2	0.9	3.8	9.6	64.1
Zn	7.7	1.2	4.8	10.7	23.4
Other cereals ^3^					
P	17.3	2.9	10.8	24.4	38.8
Co	39.0	0.0	17.1	54.9	149.8
Cu	8.2	1.1	5.7	11.9	26.0
Fe	67.3	10.5	39.1	96.4	259.8
Mn	20.4	3.9	13.8	31.1	63.0
Zn	12.7	2.4	8.7	19.0	31.7
Commercial energy supplement ^4^					
P	36.1	3.6	16.2	56.6	89.8
Co	170.4	4.9	96.9	373.6	822.1
Cu	55.5	2.0	18.8	96.1	240.4
Fe	377.9	31.0	194.4	516.0	983.8
Mn	193.8	9.2	46.9	307.8	1030.8
Zn	72.4	2.9	13.3	123.5	246.7
Soy products ^5^					
P	10.9	1.2	7.4	14.7	27.0
Co	13.4	1.6	10.4	21.1	37.8
Cu	6.7	1.0	4.6	9.4	17.2
Fe	46.8	8.2	31.3	63.5	123.5
Mn	13.0	1.2	9.2	18.2	36.2
Zn	6.2	0.8	4.2	8.2	15.7
Commercial protein supplement ^6^					
P	19.9	2.3	11.2	30.8	117.7
Co	137.6	0.0	79.8	212.0	571.8
Cu	46.3	3.3	27.0	75.6	368.7
Fe	262.9	21.5	158.0	356.5	2120.5
Mn	135.0	8.8	82.9	227.7	1344.6
Zn	57.3	3.4	33.4	92.8	773.4
Minerals and vitamins ^7^					
P	10.8	0.9	5.5	18.4	47.9
Co	98.8	6.0	50.8	180.7	382.7
Cu	43.5	1.6	22.7	81.9	182.0
Fe	204.2	11.5	113.9	389.5	1180.1
Mn	120.8	5.3	67.5	233.4	601.2
Zn	49.8	1.6	24.8	94.5	242.9

^1^ Herds using total mixed ration were not included in this analysis, due to missing information. Herds not including a feed category in their ration were excluded from the analysis of this given feed category. ^2^ Forage category included mixed grass and legume silage, corn silage, and mixed hay. ^3^ Oat, barley, wheat, and mixed cereals included. ^4^ Defined as Non-fiber carbohydrates >30% of DM. ^5^ Only 4 different samples were analyzed in this category. ^6^ Defined as CP >30% of DM. ^7^ Commercial blend. For some herds, vitamins and minerals were included in the commercial energy or protein supplements.

**Table 4:** Characteristics of cows and mineral recommendations from three different references ^1^ according to day-in-milk categories.

Items	Days in Milk Category				SEM	*p*-Value
	≤21	Between 22 and 80	Between 81 and 199	≥200		
Days in milk	12.5 ^d^	50.9 ^c^	140.7 ^b^	291.3 ^a^	1.7	<0.0001
Estimated body weight ^2^ (kg)	673 ^b^	662 ^b^	669 ^b^	692 ^a^	3	<0.0001
Milk yield (kg/day)	34.5 ^b^	39.1 ^a^	33.9 ^b^	25.4 ^c^	0.6	<0.0001
Fat-corrected milk ^3^ (kg/day)	36.3 ^a^	38.0 ^a^	33.6 ^b^	26.4 ^c^	0.6	<0.0001
Milk fat (%)	4.39 ^a^	3.83 ^c^	3.99 ^b^	4.34 ^a^	0.04	<0.0001
Milk protein (%)	3.39 ^b^	3.00 ^d^	3.26 ^c^	3.57 ^a^	0.02	<0.0001
Milk lactose (%)	4.50 ^c^	4.65 ^a^	4.59 ^b^	4.51 ^c^	0.01	<0.0001
Predicted DMI ^4^ (kg)	16.8 ^c^	22.9 ^b^	24.8 ^a^	22.6 ^b^	0.2	<0.0001
NRC recommendations						
P ^5^ (% of DM)	0.45 ^a^	0.38 ^b^	0.34 ^c^	0.32 ^d^	0.00	<0.0001
Co ^6^ (mg/kg of DM)	0.11	0.11	0.11	0.11	-	-
Cu ^5^ (mg/kg of DM)	15.4 ^a^	11.5 ^b^	10.2 ^c^	11.4 ^b^	0.0	<0.0001
Fe ^5^ (mg/kg of DM)	22.2 ^a^	17.0 ^b^	13.8 ^d^	14.3 ^c^	0.0	<0.0001
Mn^5^ (mg/kg of DM)	19.7 ^a^	14.6 ^b^	12.7 ^d^	13.5 ^c^	0.0	<0.0001
Zn ^5^ (mg/kg of DM)	69.8 ^a^	53.1 ^b^	44.2 ^c^	41.2 ^d^	0.0	<0.0001
INRA recommendations						
P ^7^ (% of DM)	0.41 ^a^	0.36 ^b^	0.31 ^c^	0.28 ^d^	0.00	<0.0001
Co ^6^ (mg/kg of DM)	0.3	0.3	0.3	0.3	-	-
Cu ^6^ (mg/kg of DM)	10	10	10	10	-	-
Fe ^6^ (mg/kg of DM)	-	-	-	-	-	-
Mn ^6^ (mg/kg of DM)	50	50	50	50	-	-
Zn ^6^ (mg/kg of DM)	50	50	50	50	-	-
EAAP recommendations						
P ^8^ (% of DM)	0.43 ^a^	0.38 ^b^	0.33 ^c^	0.30 ^d^	0.00	<0.0001
Co^6^ (mg/kg of DM)	0.1	0.1	0.1	0.1	-	-
Cu^6^ (mg/kg of DM)	10	10	10	10	-	-
Fe^6^ (mg/kg of DM)	50	50	50	50	-	-
Mn^6^ (mg/kg of DM)	40	40	40	40	-	-
Zn^6^ (mg/kg of DM)	50	50	50	50	-	-

^a,b,c,d^ Means in the same row with different superscripts differ; *p* ≤ 0.05. ^1^ EAAP = European Federation of Animal Science; INRA = Institut National de la Recherche Agronomique; NRC = National Research Council. ^2^ Estimated using heart girth circumference. ^3^ Calculated using the NRC equation: (0.4 × milk yield) + (15 × (milk fat/100) × milk yield). ^4^ Predicted dry matter intake using equations from NRC [1]. ^5^ Obtained from NRC equations provided in supplemental Table S1 divided by an absorption coefficient and then by NRC estimated DMI. ^6^ Non-factorial approach, hence no statistical analysis performed. No INRA recommendation for Fe. ^7^ Obtained from INRA equations provided in supplemental Table S1 divided by an absorption coefficient and then by NRC estimated DMI. ^8^ Obtained from EAAP equations provided in supplemental Table S1 divided by an absorption coefficient and then by NRC estimated DMI. Abbreviations: DM = dry matter; SEM = standard error of the mean. - no statistical analysis performed.

**Table 5:** Dietary concentrations of selected minerals and the percentages of dietary concentrations relative to the recommendations according to day-in-milk categories and three different references ^1^.

Items	Days in Milk Category				SEM	*p*-Value
	≤21	Between 22 and 80	Between 81 and 199	≥200		
Dietary concentrations						
P (% of DM)	0.40 ^a^	0.39 ^ab^	0.38 ^bc^	0.37 ^c^	0.01	0.0003
Co ^2^ (mg/kg of DM)	0.61 (0.59–0.63)	0.60 (0.58–0.62)	0.59 (0.57–0.61)	0.58 (0.57–0.60)	-	0.36
Cu ^2^ (mg/kg of DM)	18.9 ^a^ (17.6–20.2)	17.5 ^ab^ (16.5–18.6)	16.8 ^ab^ (15.8–17.9)	16.5 ^b^ (15.5–17.5)	-	0.02
Fe ^2^ (mg/kg of DM)	226 (208–245)	208 (193–223)	204 (189–219)	201 (187–216)	-	0.17
Mn ^2^ (mg/kg of DM)	74.8 ^a^ (68.9–81.2)	67.8 ^ab^ (63.0–73.0)	64.9 ^b^ (60.3–69.9)	63.2 ^b^ (58.7–68.1)	-	0.02
Zn (mg/kg of DM)	91.8 ^a^	84.0 ^ab^	78.8 ^b^	76.2 ^b^	2.8	0.001
% below or above the recommendations ^3^						
NRC						
P	−11 ^c^	2 ^b^	10 ^a^	15 ^a^	2	<0.0001
Co	509	493	464	452	26	0.35
Cu ^2^	23 ^b^ (15–32)	53 ^a^ (44–63)	65 ^a^ (55–76)	50 ^a^ (41–60)	-	<0.0001
Fe ^2^	930 ^c^ (849–1019)	1138 ^b^ (1050–1234)	1396 ^a^ (1288–1511)	1497 ^a^ (1382–1620)	-	<0.0001
Mn ^2^	281 ^b^ (251–314)	367 ^a^ (333–403)	409 ^a^ (373–449)	378 ^a^ (344–415)	-	<0.0001
Zn	35 ^c^	60 ^b^	80 ^ab^	89 ^a^	6	<0.0001
INRA						
P	0 ^d^	9 ^c^	21 ^b^	30 ^a^	2	<0.0001
Co	123	117	107	102	9	0.35
Cu ^2^	89 ^a^ (76–102)	75 ^b^ (65–86)	68 ^b^ (58–79)	65 ^b^ (55–75)	-	0.02
Fe	-	-	-	-	-	-
Mn ^2^	50 ^a^ (38–62)	36 ^ab^ (26–46)	30 ^b^ (21–40)	26 ^b^ (17–36)	-	0.02
Zn	86 ^a^	68 ^ab^	58 ^b^	52 ^b^	6	0.0005
EAAP						
P	−5 ^d^	3 ^c^	14 ^b^	24 ^a^	2	<0.0001
Co	569	552	521	507	26	0.35
Cu ^2^	89 ^a^ (76–102)	75 ^b^ (65–86)	68 ^b^ (58–79)	65 ^b^ (55–75)	-	0.02
Fe ^2^	351 (316–389)	315 (286–346)	307 (278–338)	302 (274–333)	-	0.17
Mn ^2^	87 ^a^ (72–103)	69 ^ab^ (57–82)	62 ^b^ (51–75)	58 ^b^ (47–70)	-	0.02
Zn	86 ^a^	68 ^ab^	58 ^b^	52 ^b^	6	0.0005

^a,b,c,d^ Means in the same row with different superscripts differ; *p* ≤ 0.05. ^1^ EAAP = European Federation of Animal Science; INRA = Institut National de la Recherche Agronomique; NRC = National Research Council. ^2^ Geometric mean and 95% CI for log-transformed data computed as e^x^ within parentheses. ^3^ Calculated as: mineral concentrations provided by the diet minus dietary recommendation then divided by the dietary recommendation. Negative values indicate underfeeding, whereas positive values imply overfeeding. Abbreviations: DM = dry matter; SEM = standard error of the mean.

**Table 6:** Descriptive statistics on percentages of dietary mineral concentrations relative to the recommendations from three different references among the 100 dairy herds ^1^.

% Below or Above the Recommendations ^2^	Median	Centile 1	Centile 25	Centile 75	Centile 99
NRC					
P	8	−34	−3	20	64
Co	405	70	274	601	1262
Cu	52	−38	26	83	246
Fe	1346	469	998	1841	4046
Mn	372	107	278	503	1195
Zn	65	−44	34	111	384
INRA					
P	19	−28	7	33	89
Co	85	−38	37	157	399
Cu	65	−29	37	98	260
Fe	-	-	-	-	-
Mn	28	−45	4	61	248
Zn	46	−49	21	96	322
EAAP					
P	12	−31	1	26	80
Co	455	87	312	671	1398
Cu	65	−29	37	98	260
Fe	319	63	232	453	882
Mn	60	−31	30	101	334
Zn	46	−49	21	96	322

^1^ EAAP = European Federation of Animal Science; INRA = Institut National de la Recherche Agronomique; NRC = National Research Council. ^2^ Calculated as: Mineral concentrations provided by the diet minus dietary recommendation then divided by the dietary recommendation. Negative values indicate underfeeding, whereas positive values imply overfeeding.

## Data Availability

Data is contained within the article.

## Supplementary Materials

The following are available online at https://www.mdpi.com/article/10.3390/ani11051320/s1, Table S1: Equations for determining mineral requirements of dairy cows according to 3 different references.
