# Supplementary material for: Toward Precision Feeding Regarding Minerals: What Is the Current Practice in Commercial Dairy Herds in Québec, Canada?
Source: Animals (Basel). 2021 May 5;11(5):1320. doi: 10.3390/ani11051320 (PMC8148184; doi:10.3390/ani11051320)
Supplement: Supplementary file 1 [file animals-11-01320-s001.zip › animals-1173816-supplementary.pdf]

SUPPLEMENTARY FILES

Supplementary table 1. Equations for determining mineral requirements of dairy cows according to 3 different references[1]

Minerals	Requirement	Sources		
		National Research Council	Institut National de la Recherche Agronomique	European Federation of Animal Science
P (g/day)	Factorial			
	Maintenance	Fecal = 1 x DMI and Urine = 0.002 x BW	Maintenance = (0.83 x DMI) + (0.002 x BW)	Maintenance = 1 x DMI
	Lactation	Milk = 0.9 x FCM[2]	Milk = 0.9 x milk yield (L)	Milk = 0.98 x milk yield (kg)
	Pregnancy	If PregD ≥ 190, then Fetal = 0.02743 x Exp(((0.05527 – (0.000075 x PregD)) x PregD)) – 0.02743 x Exp(((0.05527 – (0.000075 x (PregD – 1))) x (PregD -1)))	If PregD ≥ 190, then Fetal = 7.38/(1 + Exp(1.9 – 5.46 x ln(40 – Weeks before calving)))	If PregD ≥ 190, then Fetal = 0.02743 x Exp(((0.05527 – (0.000075 x PregD)) x PregD)) – 0.02743 x Exp(((0.05527 – (0.000075 x (PregD – 1))) x (PregD -1)))
	Growth	Growth = (1.2 + (4.635 x ($MW^{0.22}$) x ($BW^{-0.22}$))) x (WG/0.96)	Growth = (1.2 + (4.66 x ($MW^{0.22}$) x ($BW^{-0.22}$))) x WG	Growth = (1.2 + (4.635 x ($MW^{0.22}$) x ($BW^{-0.22}$))) x (WG/1000)
Co (mg/day)	Non-factorial	0.11 x DMI	0.3 x DMI	0.1 x DMI
Cu (mg/day)	Non-factorial	-	10 x DMI	10 x DMI
	Factorial			
	Maintenance	Fecal = 0.0071 x BW	-	-
	Lactation	Milk = 0.15 x FCM	-	-
	Pregnancy	If PregD < 100, then Fetal = 0.5<br>If 100 ≤ PregD ≤ 225, then Fetal = 1.5<br>If PregD > 225, then Fetal = 2	-	-
	Growth	Growth = 1.15 x (WG/0.96)	-	-
Fe (mg/day)				
	Non-factorial	-	-	50 x DMI

	Factorial			
	Maintenance	0	-	
	Lactation	Milk = 1 x FCM	-	
	Pregnancy	If PregD > 190, then Fetal = 18	-	
	Growth	Growth = 34 x (WG/0.96)	-	
Mn (mg/day)	Non-factorial	-	50 x DMI	40 x DMI
	Factorial			
	Maintenance	Fetal = 0.002 x BW	-	-
	Lactation	Milk = 0.03 x FCM	-	-
	Pregnancy	If PregD > 190, then Fetal = 0.3	-	-
	Growth	Growth = 0.7 x (WG/0.96)	-	-
Zn (mg/day)	Non-factorial	-	50 x DMI	50 x DMI
	Factorial			
	Maintenance	Fecal = 0.033 x BW and Urine = 0.012 x BW	-	-
	Lactation	Milk = 4 x FCM	-	-
	Pregnancy	If PregD > 190, then Fetal = 12	-	-
	Growth	Growth = 24 x (WG/0.96)	-	-

Abbreviations: BW = body weight; DMI = dry matter intake; FCM = fat corrected milk; MW = mature weight; PregD = days of pregnancy; WG = weight gain

[1] P requirements for all references are based on absorbed P and absorption coefficients should be applied to get the dietary requirements. Cu, Mn, and Zn from the National Research Council are also based on absorbed minerals. Absorption coefficients of 0.04, 0.1, 0.0075, and 0.15, for Cu, Fe, Mn and Zn were used. Regarding P, absorption coefficients of 0.64 for concentrate ingredients and 0.7 for forage were used for National

Research Council and Institut National de la Recherche Agronomique recommendations, whereas a coefficient of 0.7 was used for European Federation of Animal Science recommendations.

[2] Fat corrected milk = (0.4 x milk yield) + (15 x (milk fat concentration / 100) x milk yield)
